# Correlation between the severity of apnea and hypopnea sleep, hypertension and serum lipid and glycemic: a case control study

**DOI:** 10.1007/s00405-014-3076-5

**Published:** 2014-05-15

**Authors:** Celio Fernando de Sousa Rodrigues, Amanda Bastos Lira

**Affiliations:** 1 Federal University of Alagoas, Maceió, Brazil; 2 Department of Otorhinolaryngology, Santa Casa Hospital of Alagoas, Maceió, Brazil; 3 Vasconcelos Hygia street, 193, apt. 502, Green Tip, Maceió, Alagoas CEP 57035-140 Brazil

**Keywords:** Obstructive sleep apnea, Hypertension, Dyslipidemia, Hyperglycemia

## Abstract

The purpose of this study was to evaluate the correlation between the severity of obstructive sleep apnea (OSA) and the levels of blood pressure (BP), lipids and glucose, as intermittent hypoxia increases BP, changes the oxidative balance, and can induce the formation of free radicals and atherogenesis. 32 patients were evaluated about BP during wakefulness and sleep, total cholesterol and lipids, LDL (low-density lipoprotein), HDL (high-density lipoprotein), triglycerides, glucose and polysomnography. They were divided into four groups according to the respiratory events per hour of sleep (RDI): control group (RDI < 5), Group I (RDI 5–15), Group II (RDI 15–30), Group III (RDI > 30). There was no increase in BP in groups’ cases, the verification of systolic (*p* = 0.429) and diastolic (*p* = 0.475) BP in 24 h, systolic (*p* = 0.277) and diastolic (*p* = 0.143) BP during wakefulness, and systolic (*p* = 0.394) and diastolic (*p* = 0.703) BP during sleep in the control group. When implementing the Spearman correlation test, a correlation directly proportional to the severity of the disease was not observed. Regarding the level of serum total cholesterol (*p* = 0.092), LDL (*p* = 0.242), HDL (*p* = 0.517), triglycerides (*p* = 0.947), total lipids (*p* = 0.602) and glucose (0.355), there was no statistically significant difference between groups (*p* > 0.05 for all parameters). There is no correlation between the severity of OSA and BP levels in 24 h, during daytime, during the sleep and serum levels of LDL and HDL cholesterol.

## Introduction

Obstructive sleep apnea (OSA) is the most common form of sleep-related breathing disorder [3]. Obstructive sleep apnea is a chronic disease characterized by recurrent episodes of partial or total obstruction of the upper airway during sleep (obstructive respiratory events), and is considered an important cause of morbidity and mortality. As a result of this obstruction, there is a reduction or absence of airflow, causing a consequent alveolar hypoventilation with oxyhemoglobin desaturation, and prolonged cases, the increase of carbon dioxide (PaCO_2_) [1]. Obstructive respiratory events, besides resulting in oxyhemoglobin desaturation (hypoxia), cause an activation of the central nervous system causing an awakening soon and consequent sleep fragmentation, which is the main mechanism that determines excessive daytime sleepiness [13]. Hypoxia stimulates the arterial chemoreceptors (carotid receptors) which increase the activity of the sympathetic nervous system. Therefore, patients with OSA have a heightened response of these receptors when compared with individuals not apneic [11]. The cycles of hypoxia and reoxygenation may alter oxidative balance and induce an increase of free radicals, which are important factors for pathological consequences of OSA [8].

In healthy individuals, sleep is associated with a reduction in blood pressure (BP). The physiological decrease of systolic and diastolic BP during sleep is 10–15 % compared to the levels of wakefulness [20]. With parasympathetic predominance and low serum levels of catecholamines, NREM (no rapid eye movement) is marked by a reduction in heart rate, BP, heart activity and systemic vascular resistance. During REM (rapid eye movement) sleep, however, there is an increased activity of the sympathetic nervous system, heart rate and BP, clinically important effects for those with preexisting cardiovascular disease, in addition to muscle atony which makes the upper airway more susceptible to collapse and oxyhemoglobin desaturation deeper. Hence, REM sleep is physiologically important in the OSA [13].

Sleep deprivation can also match an important risk factor for high BP. The “Cross-sectional Sleep Heart Health Study” [5] states that the chance of having hypertension was higher in those who slept <6 h per night. According to the study by Hla et al. [6], the cardiovascular consequences of OSA are frequent and the most prevalent is hypertension.

The diagnosis of OSA is required to determine the presence of recurrent apneas and/or hypopneas and/or arousal related to respiratory effort (DRER) during sleep recorded by overnight polysomnography performed in a sleep laboratory [2]. Quantifying these respiratory events per hour of sleep (RDI), the disease is classified based on severity as mild, moderate and severe [1].

The intermittent respiratory events that lead to desaturation and oxyhemoglobin to hypercapnia cause an activation of the sympathetic nervous system responses to changing barometric chemoreceptors resulting in an increase in systolic BP and consequent hypertension or exacerbation of this condition. The intermittent hypoxia may also alter the reactivity of vascular dilation and constriction, and lead to the formation of free radicals, which are highly reactive chemical molecules that react with nucleic acids, lipids and proteins, altering cellular metabolism and resulting in cell damage and atherogenesis [7, 8]. Being OSA a disease with significant systemic repercussions, the main objective of this study was to investigate the relationship between the severity of the disease with hypertension and the degree of metabolic dysfunction by measuring serum lipid and glucose.

## Methods

Fifty adult patients, aged between 23 and 83 years, 28 males and 22 females, were evaluated after acceptance of the Terms of Consent approved by the ethics committee of the University of the Alagoas State, number 1152. There was no financial support as well as conflicts of interest and off-label or investigational use.

Of the 50 patients who participated in the initial survey, 18 did not complete all of the exams or did not return the survey. Therefore, 32 patients remained in the study, of whom 20 were male, aged 23–83 years and 12 were female, aged between 41 and 76 years.

Inclusion criteria for the study were:

- Being an adult;
- Has untreated OSA (except for the control group); and
- Accepted to participate.

The exclusion criteria for the study were:

- Being a carrier of chronic cardiac, pulmonary, renal, hematologic or neurologic;
- Being in drug treatment for dyslipidemia; and
- Being in drug treatment for diabetes mellitus.

For hypertensive patients using antihypertensive medication, discontinuation of medication for a minimum of 24 h prior to the ambulatory BP monitoring was required.

The patients were divided into four groups according to the severity of the disease:

CONTROL GROUP: comprising six patients with standard RDI (less than 5/h of sleep).

GROUP I: Nine patients with a RDI between 5 and 15/h of sleep, classified as having mild OSA.

GROUP II: Six patients with a RDI between 15 and 30/h of sleep, considered to have moderate OSA.

GROUP III: Eleven patients with a greater than RDI 30/h of sleep, classified as having severe OSA.

In the first evaluation, each selected patient was questioned regarding age, personal history of hypertension, diabetes mellitus, hypercholesterolemia, and smoking habits. Weight (kg) and height (m) were measured on anthropometric scales Welmy^®^ to calculate the body mass index (BMI), and BP was checked using a manual blood pressure meter BD^®^ and stethoscope BD^®^. The equipment was placed in one arm with the patient seated. Then, the following exams were conducted:

1. Laboratory: complete blood count, serum levels of total cholesterol, LDL (low-density lipoprotein) and HDL (high-density lipoprotein), triglycerides and total lipids, fasting glucose, renal function tests (through measurement of serum urea and creatinine) and liver function through the measurement of glutamic-oxaloacetic transaminase (SGOT), glutamic pyruvate transaminase (GPT) and gamma glutamate transferase (gamma-GT).
2. Overnight polysomnography (PSG), to determine RDI, average heart rate and lowest oxyhemoglobin saturation during sleep.
3. Ambulatory blood pressure monitoring (ABPM) to evaluate the BP for 24 h, distinguishing the mean BP awake and during sleep.

### Overnight polysomnography

Polysomnography was performed in a Sleep Laboratory Service of a reference in Otolaryngology Alagoas State in Brazil, following the 10–20 International System of electrode placement cranial central (C3/A2 and C4/A1) and occipital (O1/A2 and O2/A1) to obtain the electroencephalogram (EEG). Electrodes were also fixed at the outer corner of each eye to detect eye movements, resulting in electrooculogram, and electrodes at the chin, to obtain the electromyogram. Through the electroencephalogram, electrooculogram and chin electromyogram, one can determine the sleep architecture of the individual, observing the distribution of sleep stages and the presence of recurrent arousals that fragment sleep. Electrodes were also placed on the anterior chest for electrocardiogram; electrodes in the lower limbs (at the tibialis anterior muscle of each leg) to detect possible movements of the lower limbs. For this examination, we used a digital polygraph ICELERA^®^ 26-channel model Fast-Poly 26i. The filters used for EEG and electrooculogram were low-pass 35 Hz. For the electromyogram of the chin and lower limbs, we used low-pass filters 100 Hz and 10 Hz high pass, and for the electrocardiogram (ECG), we used low-pass filters 70 Hz.

To verify the flow of air through the upper airways, temperature sensors oro-nasal (Icelera thermistor^®^) and nasal pressure (Icelera cannula^®^) were used, necessitating the presence of at least one of them for the determination of apnea and hypopnea index. The thoracic and abdominal belts were placed to check the presence or absence of respiratory effort, which allows classifying the respiratory event as obstructive, central or mixed. A position sensor was attached to the chest strap, and snore sensor was positioned parallel to the trachea in the neck. To assess oxygen saturation, pulse oximetry second finger was placed on a hand. Each patient remained from 22:00 to 6:00 at the Sleep Laboratory, being permanently accompanied by a technical expert in nursing.

### Ambulatory blood pressure monitoring

The ABPM is a diagnostic method that allows the evaluation of daytime and nighttime BP, checking possible variations even during sleep. A digital sphygmomanometer Spacelabs^®^ was positioned in one of the upper and remained so for 24 h. BP checks were carried out every 20 min during the day and every 30 min at night. Averages of systolic and diastolic total in wakefulness and sleep were evaluated.

### Analysis

For statistical analysis, we obtained the mean and standard deviation of each variable. The level of significance was set at *p* < 0.05. To compare the mean, we used the nonparametric Kruskal–Wallis. To verify the correlation between the severity of OSA with systemic levels of diurnal and nocturnal BP and serum levels of total cholesterol and its fractions (LDL and HDL), triglycerides, total lipids and fasting glucose, we used the Test Spearman correlation (Cc). The programs used were Microsoft^®^ Office Excel^®^ 2007 and SPSS (Statistical Package for Social Sciences for Windows) version 17.0. In cases where a significant difference occurred between groups, post-nonparametric Mann–Whitney test was applied, to see in which groups this difference existed. For statistically significant results, an asterisk was used to characterize them. For the results that were not significant, we used the abbreviation NS.

## Results and discussion

The results obtained were as follows:

(A) Age: the average standard deviation of age among the control subjects was 48.2 years and 16.4, respectively. In the other groups, the mean and standard deviation (SD) age were: Group I: 52.3 years, SD 12.9; Group II: 43.7 years, SD 9.0; and Group III: 49.7 years, SD 18.6 (*p* = 0.749) (Table 1).

**Table 1:** Anthropometric and blood pressure of the first assessment patients

Variables	Control		Group I		Group II		Group III		*p*
	Mean	SD	Mean	SD	Mean	SD	Mean	SD	
Age (years)	48.2	16.4	52.3	12.9	43.7	9.0	49.7	18.6	0.749
BMI (kg/m^2^)	26.1	3.3	28.3	2.8	30.0	4.0	30.9	3.3	0.075
Systolic BP (mmHg)	113.3	10.3	125.6	14.2	126.7	19.7	124.5	16.3	0.339
Diastolic BP (mmHg)	73.3	5.2	79.4	8.1	82.5	7.6	81.8	12.5	0.238

Source: author of this dissertation (2011)

Results not significant between groups

(B) Sex: male, 50 % of the control group, 55.6 % of Group I, 100 % in Group II, and 54.5 % in Group III. There was therefore a proportionality between the sexes (approximately 1:1) between the groups, except for Group II, where all subjects were male (Table 2).

**Table 2:** Information relating to sex and the presence of hypertension, diabetes mellitus, hypercholesterolemia

Variables	Control		Group I		Group II		Group III	
	*n*	%	*n*	%	*n*	%	*n*	%
Sex								
Male	3	50.0	5	55.6	6	100.0	6	54.5
Female	3	50.0	4	44.4	0	0.0	5	45.5
Hypertension								
Yes	2	33.3	3	33.3	1	16.7	6	54.5
No	4	66.7	6	66.7	5	83.3	5	45.5
Diabetes mellitus								
Yes	0	0.0	3	33.3	0	0.0	1	9.1
No	6	100.0	6	66.7	6	100.0	10	90.9
Hypercholesterolemia								
Yes	1	16.7	5	55.6	1	16.7	0	0.0
No	5	83.3	4	44.4	5	83.3	11	100.0
Smoking								
Yes	0	0.0	2	22.2	1	16.7	0	0.0
No	6	100.0	7	77.8	5	83.3	11	100.0

Source: author of this dissertation (2011)

(C) BMI: the mean and standard deviation of BMI (kg/m^2^) groups were, respectively, control group: 26.1 and 3.3; Group I: 28.3 and 2.8, Group II: 30.0 and 4.0; Group III: 30.9 and 3.3. There was not therefore a statistically significant difference between groups (*p* = 0.075) (Table 1).

(D) BP: At the first evaluation of each patient, the BP and diastolic blood obtained were also not statistically significant different between the groups (*p* = 0.339 for systolic and *p* = 0.238 for diastolic BP), the average and standard deviation of each group, respectively, for systolic BP: control group: 113.3 and 10.3; Group I: 125.6 and 14.2; Group II: 126.7 and 19.7; Group III: 124.5 and 16.3, and for diastolic BP of: control group: 73.3 and 5.2; Group I: 79.4 and 8.1, Group II: 82.5 and 7.6, Group III: 81.8 and 12.5 (Table 1).

With respect to the information from the questionnaires regarding patients’ case histories:

- Over 50 % of individuals with sleep apnea severe (Group III), reported being carriers of hypertension, whereas this percentage was lower in the other groups (Table 2).
- The presence of diabetes mellitus was reported in three patients in Group I, two males and one female, and only one patient in Group III, this male. Its occurrence was not reported in the remaining participants (Table 2).
- When asked about the occurrence of increased serum levels of total cholesterol, LDL, HDL and triglycerides, 16.7 % of control subjects reported present. The response also was positive for 55.6 % of patients in Group I and 16.7 % of patients in Group II. No patient in Group III reported this change (Table 2).
- Only 22.2 % of patients in Group I and 16.7 % of Group II patients were smokers, and the smoking habit was not reported in the other groups (Table 2).

In the analysis of the PSG, there was a statistically significant difference between the groups with respect to RDI and minimum oxyhemoglobin saturation (*p* = 0.000 and *p* = 0.004, respectively). As heart rate, there was no statistically significant difference (*p* = 0.079) (Table 3).

**Table 3:** Mean and standard deviation of the respiratory events per hour of sleep, heart rate and oxygen saturation in minimal by overnight polysomnography

Variables	Control		Group I		Group II		Group III		*p*
	Mean	SD	Mean	SD	Mean	SD	Mean	SD	
RDI (events/h sleep)	1.2	1.6	9.9	2.4	21.9	6.2	46.2	15.3	0.000*
Heart rate (bpm)	57.3	5.2	65.3	4.0	64.4	5.5	64.5	8.7	0.079
NADIR O2 (%)	89.2	5.1	86.7	3.0	83.3	5.8	75.9	9.2	0.004*

Source: author of this dissertation (2011)

* Statistically significant differences between groups regarding the RDI and minimal oxygen saturation of O2 [NADIR O2 (%)]

Post-nonparametric Mann–Whitney test was applied to see which groups had a difference. Regarding the RDI, a significant difference was seen between the control group and the other groups [control and Group I (*p* = 0.001) and control Group II (*p* = 0.004), and control and Group III (*p* = 0.001)], and also between groups I and II (*p* = 0.001) and groups I and III (*p* = 0.000) and between groups II and III (*p* = 0.001). As for NADIR O2, the significant difference occurred between the control group and Group III (*p* = 0.005), and between groups I and III (*p* = 0.003) (Table 4).

**Table 4:** Post-nonparametric Mann–Whitney test, to see which groups had significant difference

Variables	Groups		*p*
RDI	Control	Group I	0.001*
		Group II	0.004*
		Group III	0.001*
	Group I	Group II	0.001*
		Group III	0.000*
	Group II	Group III	0.001*
NADIR O2	Control	Group I	0.375
		Group II	0.128
		Group III	0.005*
	Group I	Group II	0.312
		Group III	0.003*
	Group II	Group III	0.077

Source: author of this dissertation (2011)

* Statistically significant differences between groups

There was an increase in blood pressure in the groups of cases in the verification of systolic BP (*p* = 0.429) and diastolic (*p* = 0.475) in 24 h, systolic (*p* = 0.277) and diastolic (*p* = 0.143) during wakefulness, and systolic (*p* = 0.394) and diastolic (*p* = 0.703) during sleep (Table 5). When implementing the Spearman correlation test, we did not observe a correlation directly proportional to the severity of the disease (Cc = −0.202 for systolic BP at 24 h; Cc = −0.202 for diastolic BP at 24 h; Cc = −0.185 for systolic BP during wakefulness; Cc = −0.107 for diastolic BP during wakefulness; Cc = −0.095 for systolic BP during sleep; Cc = 0.027 for diastolic BP during sleep) (Table 6).

**Table 5:** Mean and standard deviation of blood pressure in 24 h during waking and during sleep, through the MAP

Variables (mmHg)	Control		Group I		Group II		Group III		*p*
	Mean	SD	Mean	SD	Mean	SD	Mean	SD	
Systolic BP 24 h	126.7	8.8	126.0	7.6	129.2	3.8	123.6	8.9	0.429
Diastolic BP 24 h	77.7	5.9	77.8	5.7	79.2	3.8	75.6	5.4	0.475
Systolic BP awake	128.8	11.3	130.0	8.8	134.2	3.8	125.6	9.4	0.277
Diastolic BP awake	79.7	8.5	81.8	5.9	85.7	1.6	78.2	6.8	0.143
Systolic BP sleep	119.2	1.6	112.4	8.4	117.5	6.1	117.5	12.1	0.394
Diastolic BP sleep	69.7	3.2	68.6	4.7	71.7	4.1	70.8	6.8	0.703

Source: author of this dissertation (2011)

Results not significants between groups

**Table 6:** Correlational analysis (test Spearman) between the groups regarding blood pressure in 24 h, during waking and during sleep

Variables	BP 24 h (mmHg)		BP awake (mmHg)		BP sleep (mmHg)	
Correlation coefficient	Systolic	Diastolic	Systolic	Diastolic	Systolic	Diastolic
	−0.202	−0.202	−0.185	−0.107	−0.095	0.027

Source: author of this dissertation (2011)

Regarding the level of serum total cholesterol (*p* = 0.092, Cc = −0.286) of their LDL (*p* = 0.242, Cc = −0.291) and HDL (*p* = 0.517, Cc = −0.114), triglycerides (*p* = 0.947, Cc = −0.095), total lipids (*p* = 0.602, Cc = −0.012) and fasting glucose (*p* = 0.355, Cc = 0.211), there was also no significant difference between the groups (Tables 7, 8), with no correlation between their values and the severity of OSA.

**Table 7:** Mean and standard deviation of total cholesterol, LDL, HDL, triglycerides, lipids and fasting glucose

Variables (g/dl)	Control		Group I		Group II		Group III		*p*
	Mean	SD	Mean	SD	Mean	SD	Mean	SD	
Total cholesterol	212.5	19.1	221.1	37.7	218.2	59.8	187.6	31.8	0.092
LDL	138.1	21.7	145.1	33.3	138.4	50.9	116.7	26.7	0.242
HDL	47.7	11.6	49.9	8.1	41.5	10.4	46.5	12.1	0.517
Triglycerides	231.2	213.7	181.9	110.1	211.3	235.0	175.4	126.7	0.947
Total lipids	688.4	176.5	851.1	292.8	822.8	374.4	732.0	296.3	0.602
Fasting glucose	87.0	9.3	98.9	20.3	85.8	9.5	108.8	49.2	0.355

Source: author of this dissertation (2011)

**Table 8:** Correlational analysis (test Spearman) between the groups regarding total cholesterol, LDL, HDL, triglycerides, total lipids and fasting glucose

Variables	Total cholesterol (g/dl)	LDL (g/dl)	HDL (g/dl)	Triglycerides (g/dl)	Total lipids (g/dl)	Fasting glucose (g/dl)
Correlation coefficient	−0.286	−0.291	−0.114	−0.095	−0.012	0.211

Source: author of this dissertation (2011)

The OSA has a higher prevalence with advancing age. Punjabi (2008), in their epidemiological study, showed that the disease affects about 3.2 % of people aged 20–44 years, 11.3 % of those 45–65, and 18.1 % who are between 61 and 100 years, demonstrating that increasing age has nothing to do with the emergence OSA. In the present study, the mean of the population affected was above 40 years, which is consistent with their findings.

The frequency of OSA in middle-aged individuals is higher in males at a rate 2–3 times greater than in women and affects about 4 % of men and 2 % of women over 30 years [22]. This was not observed in this study because there was a proportionality between the sexes of approximately one woman to one man, except for the group of moderate apnea, where all affected individuals were men.

The disease also has a higher prevalence in individuals who are obese or overweight. In the study by Peppard et al. [15], it was established that a 10 % increase of body weight increased risk of developing the disease six times. In the present study, we observed a higher body mass index in groups of cases than in the control group, with an increase in weight proportional to the severity of the disease. Although overweight is a significant risk factor for the onset and worsening of the disease, other factors such as the presence of craniofacial abnormalities [10] and/or anatomical obstacles in the upper airway as adenotonsillar hypertrophy, big tongue [18], nasal obstruction, smoking and heredity among others [22], should not be overlooked and should be valued both in research and in the treatment of OSA.

According to the study by Logan et al. [9], OSA is highly prevalent in individuals with cardiovascular disease, especially in hypertensive population, affecting approximately 30–83 % of these. Similar prevalence study was also observed in Hla et al. [6], where approximately 40–60 % of individuals with untreated OSA had hypertension, and among hypertensive patients, about a third had associated with OSA. The same was demonstrated subjectively in this study, where more than 30 % of apneic reported mild to moderate hypertension, and this co-morbidity was even more frequent among severe apnea, affecting more than 50 % of them. However, the objective results obtained by the analysis of a BP check in the first assessment performed in the doctor, the mean systolic BP and diastolic BP in the different groups revealed no cases of BP above normal. This fact is probably to be explained by those patients who are making use of antihypertensive medication. Epidemiological studies like the Wisconsin Sleep Cohort Study [14] and Sleep and Heart Health [12] concluded that the association between OSA and hypertension is independent of other risk factors such as age, obesity, gender, and consumption of alcohol and cigarettes. Another study carried out to verify OSA as a risk factor for hypertension [7] showed that for each respiratory event per hour of sleep, increased around 1 % risk of this individual having hypertension. Hla et al. [9] showed that individuals with OSA with RDI ≥ 5/h had higher levels of BP in wakefulness and during sleep as compared to the control group. However, this did not occur in this study because there was no significant difference between the systolic and diastolic BP at first visit, and in the evaluation of BP in 24 h during waking and sleep between the groups, since there was not a correlation between disease severity and increased levels of systemic BP.

Moreover, although no significant changes occurred in blood pressure in the different groups’ cases, two aspects must be considered. The first one is in relation to the number of heart beats per minute during sleep. Although no significant differences occurred between the mean heart rate, an increase was observed in this group of cases, objectively demonstrating that patients with OSA have an increased activity of the sympathetic nervous system when they are sleeping, which favors for the emergence or worsening of cardiovascular diseases. Normally during NREM sleep, there is a drop in the activity of the sympathetic nervous system and, consequently, heart rate and BP [22]. But in individuals with OSA, there is no drop of physiological activity of this system, resulting in inflammatory and metabolic effects that bring overhead to the operation of the entire cardiovascular system [21]. The other aspect to be highlighted is the decrease in the blood level of oxygen observed by the average minimum oxyhemoglobin saturation (NADIR O2), and the difference was statistically significant between the groups, and their level is inversely proportional to the severity of the disease; the smaller the oxyhemoglobin saturation, the greater the severity of the disease.

With respect to glucose metabolism, Punjabi et al. [16] observed an increase in peripheral insulin resistance with an increase in the RDI. A RDI ≥ 5 respiratory events/hour was associated with an increased risk for impaired glucose tolerance (odds ratio 2.15, 1.05–4.38, 95 % CI) after adjustment for BMI and body fat percentage, and down 4 % in oxyhemoglobin saturation presented an odds ratio of 1.99 (1.11–3.56, 95 % CI) also for glucose tolerance. Nevertheless, there was not a significant increase in glucose level in affected individuals in this study. Li et al. [10] concluded that intermittent hypoxia may contribute to the development of hypercholesterolemia in rats, and that it increases the HDL cholesterol level, but its activity to convert a pro-inflammatory and pro-atherogenic. Girardin et al. [4] have shown that patients with OSA showed an increase in HDL dysfunctional. Naturally, Spiegel et al. [19] in their analysis, found that patients with OSA showed an increase in HDL oxidized LDL and dysfunctional, there is a strong correlation between the severity of disease and oxidative stress caused by it with the respective elevations. This was not observed in this study, since there was no significant difference between the levels of lipids in affected individuals compared to the control group, the same event that occurred in the study of Punjabi et al. [16], where the LDL and triglyceride levels were not associated with the severity of OSA. Therefore, studies to date are conflicting regarding the actual occurrence of metabolic disorders such as dyslipidemia and hyperglycemia in patients with OSA.

## Conclusion

There is no correlation between the severity of OSA and elevated blood pressure in 24 h, during wakefulness and sleep, as well as elevated the serum levels of total cholesterol, LDL, HDL, triglycerides, and fasting glucose. However, the duration of OSA may be an important factor in the onset or worsening of hypertension and metabolic disorders.
